# The development of a lifestyle modification mobile application, “Health for You” for overweight and obese breast cancer survivors in Korea

**DOI:** 10.4069/kjwhn.2021.09.14

**Published:** 2021-09-30

**Authors:** Su-Jin Seo, Ju-Hee Nho, Youngsam Park

**Affiliations:** 1 Department of Nursing, Kunjang University College, Gunsan, Korea; 2 College of Nursing, Jeonbuk Research Institute of Nursing Science, Jeonbuk National University, Jeonju, Korea; 3 Department of General Surgery, Presbyterian Medical Center, Jeonju, Korea

**Keywords:** Breast neoplasms, Life style, Obesity, Program, Survivors

## Abstract

**Purpose:**

This study aimed to develop a lifestyle modification (LSM) mobile application based on the Android operating system for overweight and obese breast cancer survivors (BCS) in Korea and evaluate its usability.

**Methods:**

The content analysis, design, development, implementation, and evaluation of the LSM intervention mobile application for overweight and obese BCS was conducted by identifying survivors’ needs, searching the literature, and reviewing existing mobile applications. The survey was conducted from June 1 to December 28, 2020 at Jeonju city, Korea.

**Results:**

The mobile application for BCS included dietary and exercise information, weight logs, as well as distress and daily achievement check. It also included information and videos on the prevention of breast cancer recurrence and used a communication bulletin board. Expert and user usability evaluation of its content and functions confirmed that it was appropriate and satisfactory for overweight and obese BCS.

**Conclusion:**

This LSM mobile application developed for overweight and obese BCS was found to be appropriate for use. It can be applied for further study of effectiveness on improving their health and maintaining a healthy lifestyle, to ultimately improve quality of life.

## Introduction

Studies on breast cancer have developed diagnostic methods for early diagnosis and treatment due to high screening rates [1]. Specifically, breast cancer survival rates continue to increase in Korea, reaching a 5-year relative survival rate of 93.3% [2], higher than that of other cancers. The incremental increase in the number of cancer survivors has led to a shift to adopting a chronic disease management approach to cancers [3], and as a high number of young women are diagnosed with breast cancer, their long-term quality of life should be considered [4]. The main risk factors of cancer include obesity, drinking, eating, and smoking, and many women are overweight and obese when diagnosed with breast cancer [5]. Studies have shown that healthy eating habits, maintaining normal weight, regular physical activity, and smoking abstinence can reduce breast cancer risk by 34% [6].

Breast cancer prevention is an important goal that includes physical activity and dietary management because being overweight and obese contribute to higher cancer incidence and recurrence rates [7]. Studies of cancer survivors show that those who maintained a healthy weight, regularly practiced physical activity, abstained from drinking, and ate well-balanced foods, had lower mortality rates [8]. Moreover, breast cancer survivors (BCS) who consumed more than five vegetables and fruits a day and walked more than 30 minutes a week had a higher survival rate than those who ate red meat-based diets and were physically inactive [9-11]. However, a comparative study of the characteristics of cancer patients and survivors found that the former had a high frequency of drinking and a low rate of cancer screening, which worsened their lifestyles after treatment [11]. According to a study that evaluated obesity among BCS in Korea, 67.4% were overweight, and 48.4% were obese with low physical activity [5].

BCS reported that they have to deal with the after-effects of treatment, and a variety of physical, social, and psychological problems which need attention and support. Physical problems included pain, lymphedema, fatigue, and changes in sexual function. Psychological problems were anxiety, depression, and distress, leading to a poor overall quality of life [12]. Thus, effective management intervention strategies for BCS need to be developed [13].

Services using the internet and mobile applications are being transformed according to consumer expectations for high-level medical services [14]. These include applications related to health promoting programs and healthcare education and ongoing studies are assessing their usability [15]. Education and management using mobile applications continue to grow because of their convenience, excellent accessibility, and the advantages of unrestricted mobility [16]. Moreover, it is used not only in cancer but also in various health issues such as stroke, brain damage, and dementia because individual characteristics and customized health education can be low cost and effort. Specifically, these applications have been applied to BCS to reduce depression and improve quality of life [17]; studies show that using these to maintain a healthy lifestyle increased motor motivation and physical activity [18]. However, these two studies have not been tailored to the needs of overweight or obese BCS.

Therefore, the purpose of this study was to develop a mobile lifestyle application for overweight and obese BCS and confirm the validity of the application. The application seeks to promote the health of BCS by providing information on the importance of a healthy lifestyle (e.g., diet and exercise) and ways to attain it.

## Methods

Ethics Statement: This study was approved by the Institutional Review Board of the Presbyterian Medical Center (2020-05-021). Informed consent was obtained from the participants.

### Study design

**Figure 1.**
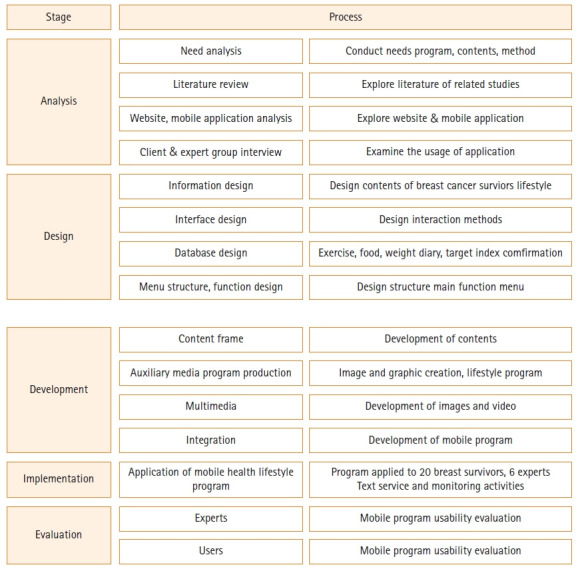
Process of the mobile application development

This methodological study aimed to develop a lifestyle modification (LSM) mobile application for overweight and obese BCS and to evaluate the usability of the developed application. The LSM mobile application was developed according to the ADDIE (Analysis, Design, Development, Implementation and Evaluation) model [19] (Figure 1). Its advantages include an ongoing review of objectives in the development process, interrelationships of elements, and feedback and modifications based on real-world experience [20].

#### Analysis phase

We performed need analysis, literature review, web site and mobile application review, and group interview in this phase. For the need assessment, 20 BCS were recruited via convenience sampling from the breast cancer outpatient clinic of the Presbyterian Medical Center in Jeonju, Korea. Inclusion criteria were those who aged 19 to 65 years, 1 year after the end of treatment, and have not been diagnosed with a psychiatric disease. As the exclusion criteria, those who had metastasized or recurred breast cancer to other sites and those without a spouse were excluded. The needs assessment survey was conducted from June 1 to 30, 2020, to determine the functional and needs requirements for the LSM mobile application. Surveys and interviews were conducted in the outpatient counseling room, and took about 20 minutes.

The contents of the applications were organized using literature reviews and other mobile applications. We reviewed the contents from following sites: The KISS (Korean Studies Information Service System), NDSL (National Digital Science Library), CINAHL (Cumulative Index to Nursing and Allied Health Literature), RISS (Research Information Sharing Service), EMbase, Pubmed, and Cochrane Library. The main content and effects in the exercise and diet of BCSs were identified and applied in the mobile application using BCSs. We searched for keywords such as “BCS and exercise,” “overweight BCS and lifestyle program,” “BCS and diet,” “BCS and immune function,” “BCS and mobile application,” “BCS and partner,” “BCS and couple,” and “BCS and marital.” As a result of the literature search within the last 10 years, a total of 1,027 papers were searched. For domestic literature search, “BCS” OR “breast cancer” AND “exercise” AND “diet” AND “lifestyle” AND “mobile application” AND “smartphone” AND “marital” AND “psychological” keywords were applied in RISS and KISS. The search expression used the MeSH (Medical Subject Headings) expression in Pubmed ((“BCS” [Mesh] OR (“C”[TW] OR “Obesity” OR “Overweight”[TW])) AND “Exercise”[Mesh] AND “Diet”[Mesh] AND “Mobile application” AND “Partner” AND “Couple” AND “Marital” . The search was performed using CINAHL headings (BCS OR Breast cancer) AND (Diet OR Exercise OR Lifestyle OR Mobile application OR Couple OR Partner OR Marital OR Psychological. The English association used keywords such as “breast cancer,” “exercise,” “lifestyle,” “diet,” “overweight,” “obesity,” and “mobile applications,” to conduct searches. The contents were analyzed after searching for “living habits” and “breast cancer” on mobile applications related to lifestyle management of BCSs in the Google Play Store of Android mobile phones. Using the combined search terms, a total of 1,027 studies were identified, including 104 studies from Korean databases (DBs) and 923 studies from foreign DBs. As a result of the keyword search results, the number of papers on “body exercise for BCSs” was 430, “the diet of BCSs” was 172, “the number of psychological searches of BCSs” was 262, “mobile program” was 39, and “the number of spouse searches” was 124. Among the searched papers, nine physical exercise programs, four psychological programs, three dietary programs, five mobile programs, and 10 spouse programs were selected for BCSs.

We searched and analyzed existing mobile lifestyle applications for BCSs to organize the configuration items of primary lifestyle applications. It was based on the purpose and content of each item. The searched application types were ‘breast cancer by second doctor,’ ‘Bravo,’ ‘Pink Touch,’ ‘Overcoming Breast Cancer,’ ‘Pink Avatar,’ ‘Dear Mamma fights breast cancer,’ ‘Owise-Breast cancer support,’ ‘Breast cancer Healthcare,’ and ‘Outcomes4me Breast cancer care,’ etc. Based on the literature review, open questionnaires for users and experts were developed and data were collected with specific details. The survey consisted of 25 questions to identify overall healthy LSM requirements such as educational experience and satisfaction, and the need for a correct lifestyle, exercise, and diet. The analysis of mobile usage consisted of eight questions (e.g., comprising model, duration, purpose of use, as well as user experience and access to the application). The primary lifestyle application components and related contents were modified and supplemented, and interviews were conducted using open questionnaires to check the appropriateness and new ideas of the secondary components. Five experts in their field (one breast cancer surgeon, two nursing professors, and two breast cancer nursing specialists) were surveyed using open questionnaires to identify the appropriateness and new ideas for primary components. Based on the primary composition, we derived questions for the expert consultation, and the detailed questions were as follows: “What additional items are required for overweight and obese BCSs’ mobile applications?”; “What are the most important lifestyle factors of overweight and obese BCSs?”; “What should researchers be aware of while developing mobile applications for overweight and obese breast cancer patients’ lifestyles?” We identified the importance of lifestyle management for overweight obese BCSs, the challenges in current exercise methods and dietary control methods, the reasons for continuous failure, and the essential aspects to include in BCS mobile applications.

For analyzing professional lifestyle requirements, we performed interviews with five experts. The first question was, “What do you think is the most important aspect of overweight and obese BCSs’ lifestyles?” The second question was, “What do you think is needed in their mobile applications?” The third question was, “What do you think should be considered carefully by researchers when developing these applications?”

For analyzing of the mobile usage status of the subjects, the mobile models, usage period, application usage experience and purpose, disease-related information search, and satisfaction with the results were identified.

#### Design phase

In this phase the application was created with the content identified in the analysis phase and structure and requirement functions were determined. The definition and flow chart of the application were derived by referring to prior studies and research reports of overweight and obese BCSs. The menu of the mobile application is diverse and configured to conveniently find submenus. There are different menus for BCSs and their spouses and the administrator. The former is divided into information on preventing recurrence of breast cancer, exercise, diet, space for communication with spouses, bulletin boards for communication with administrators, and application introductions. It consists of layouts that allow overweight and obese BCSs and spouses to increase readability. Journals are included to record every meal, and exercise, time, type, and strength on daily basis to check the daily weight management using body mass index. It was configured to enter its target index daily, and allowing it to be verified. The formula, exercise and weight log, target index, and bulletin board information entered by BCSs is stored on the company server. The input, timing, and the number of accesses can be checked through it.

The menu structure and key functions are designed with main categories: login and introduction, expression, exercise, distress, intimacy with spouse, and target index confirmation boards. A design with user experience or user interface is applied, which is easily accessible and available to BCSs. The application based on the operating system of Android-based Google open platform is managed using member, exercise diary, expression, target index, and other information databases. It is designed using minimal tables to implement applications.

#### Development phase

We commissioned a company specializing in mobile application production with design experts and programmers. To develop the application, we used the Android system. The menus used by BCS were divided into a diet diary, exercise diary, weight diary, and goal achievement level, which were to be entered each day; as well as communication space with spouse, bulletin board, and introduction. The layout was organized to improve the readability of BCS and spouses. Food log, exercise log, weight log, target index, and bulletin board information entered by BCSs are stored on the company server, and the input contents and the access time and frequency of the subjects can be viewed through the administrator computer through the company server.

#### Implementation phase

Pilot implementation of the developed LSM application was done at this stage. Six experts (one breast surgeon, two nursing professors, two breast cancer nursing specialists with more than 3 years of experience, and one doctoral expert in computer science with experience in creating mobile applications for patients) were invited. Also, 20 BCS (including 10 women who had participated in the needs assessment) were recruited for usability testing. The same inclusion and exclusion criteria were applied, with the additional inclusion criteria of BCS who could install the Android-based application. Participants were between 19 and 65 years.

Participants were asked to install the ‘Health for You’ application on their cell phones and sign up for membership, and were approved by the administrator (researcher). Both groups were required to use the application for two weeks from December 14 to 28, 2020.

#### Evaluation phase

After 2 weeks of use, experts and users evaluated the aesthetic, informational, and lifestyle aspects of the LSM mobile application.

Measurement: The following instruments were used, after obtaining permission for use from the original authors: (1) Usability evaluation of experts: The expert usability assessment tool developed by Kim [21] was used to evaluate mobile healthcare applications. The three main factors are content, interface design, and technology with 23 items including objectivity, understanding, consistency, accuracy, vocabulary accuracy, design suitability, and security. Each item on the 4-point Likert scale is measured from 0 to 3, with 0 as the lowest and 69 as the highest; the higher the score, the better usability the application. This mobile application evaluation tool was developed with Cronbach’s α of .91 [21] and was .93 in this study. (2) Usability evaluation of users: The user version of the Mobile Application Rating Scale [22] was used for user evaluation of usability. There are six factors with 26 items in total: three on aesthetic factors, four on information, five on participation, six on perceived influence, four on functionality, and four on subjective quality assessment. Each item on the 5-point Likert scale is measured from 1 to 5, the higher the score, the better usability the application. This tool was developed as a mobile application evaluation tool with Cronbach’s α=90 [22], and was .94 in this study.

Procedures: The survey was conducted for 15 minutes in the breast outpatient interview room. Based on the evaluation results of experts and users, the revisions were made: Thus, the mobile application, “Health for You” was finalized.

#### Analysis of data

The data were statistically processed using the IBM SPSS for Windows, ver. 24.0 (IBM Corp., Armonk, NY, USA). Descriptive statistics (percentages, means, and standard deviations) were done for general characteristics (age, spouse age, education level, occupation, religion, monthly income, body mass index, stage, type of treatment, exercise activity, number of exercise, and exercise time), and the usability responses of experts and users.

## Results

### Analysis phase findings: needs of participants and experts, content, and mobile status

The mean age of 20 BCS who participated in the survey was 51 years and the mean body mass index was 26.8 kg/m^2^ (Table 1). Most of the patients were unaware of 85% of the education on lifestyle and wanted to receive 90% of the education on LSM. Most of the desired lifestyle education methods were mobile applications (50%), followed by 1:1 education (30%). Community activities for mobile use were higher than 90%, followed by information searches at 75%, and 90% wanted to use the applications (Table 2).

Analysis of 35 studies and 10 applications on the BCS lifestyle program consisted mainly of breast cancer definition, classification, symptoms, diet, exercise, and methods for managing after-effects. Mostly the basic diet, exercise, breast cancer symptom management, and proper healthcare methods are presented, which are also included in the importance of managing weight and obesity, exercise methods, and diet tables (Supplementary Table 1).

Expert lifestyle needs were examined through open-ended questions, the importance of lifestyle management and weight loss in most BCSs is linked to the need for education and information.

### Design phase findings

Determination of the structure and requirement function, definition, and flowchart of the application led to designing main menus and submenus. The main menus included application introduction, diet, exercise, distress, spouse and intimacy, target index verification, bulletin board, and archives. Cool diet, diet rules for weight loss, low-calorie food selection, energy intake determination, calorific comparison, diet diary preparation, food calorie table designed by exercise method, weight loss success strategy, aerobic exercise type, muscle training guide, weight log preparation, and distress self-test are included. Submenus were created for announcements, frequently asked questions, and breast cancer-related archives.

The components of the application were established, and the details and methods of the application were designed (Figure 1). The items developed in this study consist of six categories: dietary management, exercise management, distress management, spouse participation, goal index verification, and data room.

First, dietary management conveys information about diets that would make the subject aware of the importance of lifestyle and have the correct dietary habits. Clicking on “diet” on the menu provides information on topics such as breast cancer and obesity, lifestyle definition, the importance of a healthy lifestyle, breast cancer, weight-loss method, dietary control method, and the calorimetry table by food and by cooking method. A daily dietary diary was prepared to display the results on the calendar to display the number of meals consumed every day which can help identify the importance of proper diet management.

Second, clicking on “exercise” shows videos on exercise methods, weight-loss exercises and strategies, brain yoga, aerobic exercises, stretching guides, muscle exercises, and theoretical information. Daily exercise logs include the type, time, and the number of steps, and the results are linked to the calendar.

Third, the submenu on distress management consists of the definition, self-test, and ways to overcome it. The definition provides an accurate understanding of the distress experienced by cancer survivors, and the self-test items provided an index of distress with specific distressing self-test items such as physical, real-life, family, emotional, spiritual, and religious problems. Maintaining a healthy life, actively managing physical symptoms, building positive experiences, getting help from experts if necessary, and talking to people helps in overcoming stress.

The option, “Spouse and Intimacy” in the fourth menu consists of compliment stickers, making healthy food, doing aerobic exercises together, and creating happy canvas frames. It is intended to increase the intimacy of couples by listing activities that they can do together.

Fifth, the goal index verification was including goal of program. The program recommended to consistently eat three meals a day for more than 20 minutes with many vegetables or fruits, and drink sufficient amounts of water. Specifically, the goal is to “walk more than 10,000 steps a day” and “stretching more than twice a day” to the point of sweating for more than 30 minutes. The goal index is checked and recorded in the diary every day. To relieve stress, there are checkboxes with phrases “satisfied with myself today,” “happy day,” “went to bed comfortably without worrying,” and “sleeping well without waking up in the middle.” The options, “walking with my spouse,” “stretching with my spouse,” and “sending compliment texts once a day” are configured to check the relationship with the spouse and to fill out the daily target index in the diary. This space supports interaction with the spouse, and increases the time spent on exchanging messages and providing active support.

Finally, the data room comprised information on BCSs such as sex life, and lymphedema definition and prevention. It also included “Q&A,” “Notice,” and “Question Room.” In the question room on the main screen, opinions are exchanged, and questions are answered and supported by nurse.

The application has a variety of menus and is designed to be convenient for finding submenus. The two menus are for BCSs and administrators. For the former, it is divided into application introduction, diet, exercise, information space for breast cancer patients, spouse and communication space, and bulletin boards for communication with administrators. The other is for the administrator to check the information entered by the patient. The layout is configured to increase readability and easy access to subjects. Dietary journals are designed to record daily diet as well as exercise methods time, and exercise intensity. The weight log was automatically calculated and the weight goal management was verified every day. It was also configured to enter its daily target index, and allowing it to be verified. The dietary, exercise, weight, target index, and bulletin board information are individually stored on the company server, and the input and the timing and number of accesses of the target person can be viewed through the server using the administrator’s computer.

In addition, for designing the application, we used a minimum number of tables, and used a design that is linked to the patient’s ID at the time of registration. Information regarding food, exercise, and weight diaries as well as target index and “distress checks” were directly added and stored by the user.

### Development phase findings

Based on the results of the analysis and design phases, this LSM application was created and named as “Health for You” application after repeated refinement of the production and LSM process. We used the Android Java Development Kit (JDK) 1.8.0, and Android Studio as development environments, with a minimum operating system of Android 4.3 and a maximum of Android 9.0.

### Usability testing findings

Based on feedback from experts and users, the following revisions were made: As for the revised and revised contents, the diet and exercise log parts were moved to the screen, and exercise methods for each exercise type were added. A connection item was added so that the subject and spouse in the membership registration column match. An explanation of the lymphedema exercise method of BCSs and a video were additionally inserted.

For expert usability, the average evaluation score was 3.33±0.51 out of 4; “Application icon arrangement and design uniformity” showed the lowest score in interface design sub-factor consistency with 2.67±0.51; the application provided reliable healthcare information and familiar words. Among them, nine items were derived with two contents, three interfaces, and one security, with an average score of 3.33±0.51 points or less, and nine suggested revisions. Moreover, the total score was 65.66±6.98 and the patient LSM application was evaluated positively. Based on this, eight opinions were modified, except for duplicated opinions, and nine items received an average of 3.33±0.51 points or less in the evaluation of the mobile healthcare application (Table 3).

Results of usability testing with the 20 overweight and obese BCS found that the lowest-scoring question was from the participation aspect, “Are the applications interesting to use?” In contrast the highest-scoring questions were “Was it easy to learn how to use an application?” and “How accurately and quickly do the functions and components work?” from the functional area. All 26 questions scored more than 3 points, 24 scored more than 3.5 points, and eight scored more than 3.8 points (Table 4).

**Figure 2.**
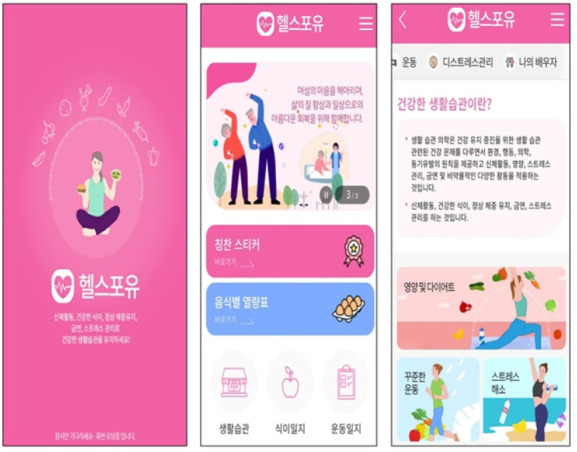
The “Health for You” lifestyle modification mobile application.

The final interface of the developed LSM mobile application, “Health for You,” is shown in Figure 2.

## Discussion

This study aimed to develop a LSM mobile application for overweight and obese BCS and test its usability with experts and BCS. According to a survey on lifestyle-related needs of overweight obese BCSs, educational experience was 85% or more, information acquisition methods were over 50% higher through mobile, and support from medical staff was 15% lower. After breast cancer treatment, many people opined it was difficult to maintain a healthy lifestyle. The demand for healthy lifestyles was high, but there was a lack of information regarding healthy lifestyles and support from medical staff was also limited, which is consistent with the results of a previous study [18]. Therefore, it was confirmed that active intervention and support from the medical staff related to lifestyle and improvement of BCSs were required. BCSs have to manage important factors that impact their healthcare, along with proper weight maintenance after chemotherapy, hormone therapy, and radiation treatment [23]. Weight gain is attributed to hormonal changes after treatment, changes in body composition, and a decrease in health behavior. Proper physical activity and healthy dietary habits are not followed, which is an important problem that the application is aiming to address [24].

After checking the participant’s needs, application content was customized for the user. The importance of a healthy lifestyle management program for BCSs was confirmed. This was also used in this study based on the results provided by prior literature that demonstrated that recording diet, exercise, and weight through self-monitoring helps maintain healthy behavior [25].

Information on the prevention and risk factors of breast cancer has been used on official websites such as the Korean Cancer Center and the Korean Breast Cancer Society [2], and it has sought to improve knowledge by providing health information. The contents and validity of the mobile application were verified by experts. With the recent development of information and communication technology, mobile applications have been developed and used in various ways without restrictions of time and place. Health promotion and management education programs using applications are actively developed and utilized in healthcare [26]. Mobile applications have been reported to be helpful to healthcare by increasing knowledge of disease and allowing better monitoring for chronic patients [27]. Since lifestyle healthcare is considered more important for cancer survivors, such as those with chronic diseases, it can be utilized appropriately to enhance their effectiveness [5]. Specifically, BCSs in their late age of 40s and early 50s usually have to return to work or do housework after acute treatment, making it difficult to attend arbitration programs at a certain time and place [18]. Currently, in an environment where collective infections are feared due to COVID-19, mobile applications, regardless of the time and place, can be expected to be more effective in helping people throughout their lives than physical interactions. Prior studies using mobile applications reported that increased motor motivation, physical activity, and vegetable intake resulted in positive health behaviors [18]. In this study, over 50% preferred mobile applications as a desired lifestyle education method and 90%, mobile users preferred 75% information retrieval and 90% community activities, which is expected to be highly utilized.

In the expert usability assessment of the developed application, most areas received more than three out of four. This seems to reflect the results of conducting lifestyle needs and environmental analysis using systematic literature reviews and researching the needs of expert groups, as well as mobile applications for breast cancer patients. According to the experts’ usability evaluation, the information availability of medical professionals was high, which used professional materials from the Korean Breast Cancer Society [2] and reflected expert opinions. The application icon arrangement and design uniformity were the lowest, and similar results were shown in a study using the same tool [28]; design experts participated in problems caused by the limited screen size of mobile devices, but it needs to be corrected to improve design satisfaction. Moreover, security protocol suggestions for personal information are low, and additional information is expected with notifications from server. From the user’s perspective, the average score was above three out of five and “ease of use” was the highest, with a mean of 4.00±70 points. However, in terms of entertainment and relevance, information provision and method of living, such as exercise and diet, scored the lowest compared to existing applications; the content in the application developed by this study can feel boring compared to applications such as games. Therefore, it is necessary to develop more interesting content to increase the expected effect.

In BCS, the spouse has a significant role as a primary health provider, indicating a similar level of psychological stress and quality of life, and that their adaptation and support can have a positive effect [29,30]. In view of this, a menu is inserted to gain the interest and support of the spouse. The partners participated and created a space to communicate with each other to increase the level of intimacy through a lifestyle intervention mobile application [4,27]. A compliment sticker and ways to exercise and make healthy food together were added. This is expected to help increase the intimacy of BCSs with their spouses. It also allows users to interact with experts through breast cancer information boards, Q&A, and announcements.

The limitations of this study are as follows: first, in this study, the requirements of breast cancer spouses were not identified and reflected, so it is thought that strategies to increase effectiveness will be needed in future studies. Also, it is necessary to think about how to encourage spouse participation after program development. Second, this study is difficult to generalize because it only focused on BCSs from one region.

In conclusion, the LSM mobile application for overweight and obese BCS, “Health for You” was found to be appropriate in terms of content, design, function, and quality according to its usability evaluation by experts and users. Subsequently, the LSM mobile application can be used with overweight and obese BCSs, and we suggest a study to verify its effectiveness and identify potential clinical significance.

## Figures and Tables

**Table 1.** General characteristics (N=20)

Variable	Categories	n (%) or mean±SD
Age (years)		51.05±7.05
	<40	1 (5.0)
	41–50	9 (45.0)
	≥50	10 (50.0)
Spouse age (years)		54.50±9.24
	<40	1 (5)
	41–50	6 (30)
	≥50	13 (65)
Educational level	<High school	2 (10)
	High school	12 (60)
	≥College	6 (30)
Occupation	Yes	16 (80)
	No	4 (20)
Religion	Yes	18 (90)
	No	2 (10)
Monthly income (Korean won)^†^	<1.9 million	4 (20)
	2–3.9 million	6 (30)
	≥4 million	10 (50)
Body mass index (kg/m^2^)		26.8±3.1
Cancer stage	1	5 (25)
	2	8 (40)
	3	7 (35)
Type of treatment	Surgery	2 (10)
	Surgery+chemotherapy	3 (15)
	Surgery+RT	5 (25)
	Surgery+chemotherapy+radiation therapy	10 (50)
Exercise activity	Yes	13 (65)
	No	8 (40)
Exercise frequency	Everyday	1 (7.6)
	5–6 times a week	2 (15.3)
	3–4 times a week	7 (23)
	1–2 times a week	3 (54.1)
	30 minutes a week	1 (7.8)
Exercise time	30 minutes–1 hour	6 (46.1)
	1 hour–2 hour	6 (46.1)

† One million Korean won=roughly 900 US dollars.

**Table 2.** User lifestyle-related characteristics and user mobile usage (N=20)

Variable	Categories	n (%)
Lifestyle education experience	Yes	3 (15.0)
	No	17 (85.0)
How to acquire lifestyle related information	Medical person	3 (15.0)
	Professional text book	2 (10.0)
	Internet via personal computer	4 (20.0)
	Mobile phone	10 (50.0)
	Family, friends and acquaintances	2 (10.0)
The necessity of lifestyle education	Vary needed	11 (55.0)
	Need	5 (25.0)
	Usually	4 (20.0)
Desired lifestyle education method	Mobile application	10 (50.0)
	1:1 education	6 (30.0)
	Education book	4 (20.0)
Intention to use the application	Yes	18 (90.0)
	No	2 (10.0)
Mobile device	Android	18 (90.0)
	Apple	2 (10.0)
Mobile usage period (years)	<3	8 (40.0)
	≥3	12 (60.0)
Mobile app experience	Yes	20 (100)
	No	0 (0)
Mobile purpose	Phone use	20 (100)
	Games	4 (20.0)
	Information retrieval	15 (75)
	Education learning	6 (30)
	Community activities	18 (90)
	Shopping	5 (25)
	Other	4 (20.0)
Disease-related information search	Yes	18 (90.0)
	No	2 (10.0)
Satisfaction with information search results	Yes	11 (55.0)
	No	9 (45.0)

**Table 3.** Expert usability evaluation (N=6)

Top evaluation factor	Sub-factor evaluation	Evaluation question	Mean±SD
Contents	Accuracy	Reliability of application information	2.83±0.40
		Clarity of application information	3.00±0.00
	Understanding	Understanding of health care information	3.00±0.00
		Use familiar words from application terminology	3.00±0.00
		Easy level of application information	3.00±0.00
	Objectivity	Expertise in health care information	3.17±0.40
		System and specificity of health care information	2.83±0.75
		Authoritative authority	3.00±0.00
		Information provision of medical professionals	3.17±0.40
Interface design	Consistency	Consistency of color, arrangement, and expression method	3.17±0.40
		Application icon arrangement and design unity	2.67±0.51
		Group app icons	3.17±0.40
	Design	Logic of content placement	3.00±0.00
	Suitability	Meaning clarity of icons	3.00±0.63
		Application text size and font comprehension	2.83±0.75
		Visual comfort	3.00±0.00
		The degree of visibility of the application structure	3.00±0.00
	Vocabulary	Brevity of application text	3.00±0.63
	Accuracy	Accuracy of application text	2.83±0.75
		Grammatical accuracy of application phrases	3.00±0.00
		Presentation of information on personal information protection	3.00±0.00
		Suggestion of security policy for personal information protection	2.83±0.40
Skill	Security	Description of security system	3.00±0.00
Total mean			3.33±0.51

**Table 4.** User usability evaluation (N=30)

Domain	Item	Mean±SD
Participation	Entertainment	3.10±0.88
	Interest	3.33±1.15
	Personalization	3.19±0.87
	Interaction	3.38±0.80
	Appropriateness for the subject	3.71±0.78
Functionality	Performance	3.90±0.76
	Ease of use	4.00±0.70
	Screen movement	3.57±0.67
	Finger gesture function	3.86±0.72
Aesthetics	Layout	3.86±0.65
	Graphic	3.86±0.65
	Visual effect	3.71±0.71
Information	Information quality	3.76±0.62
	Amount of information	3.90±0.62
	Visual information	3.24±0.94
	Reliability of data sources	3.71±0.64
Subjective quality	Willingness to recommend the app to others	3.76±0.62
	Intention to re-use for the next 12 months	3.24±0.94
	Intention to purchase the app	3.24±0.94
	If you give the app a total score	3.67±0.79
Lifestyle aspect	Raising awareness of the importance of lifestyle	3.71±0.56
	Knowledge and understanding of lifestyle	3.52±0.51
	Change in my attitude toward lifestyle	3.67±0.57
	Improving intention/motivation for lifestyle	3.62±0.66
	Degree of help for lifestyle	3.57±0.67
	Improving my lifestyle behavior	3.57±0.59
	Total mean	3.60±0.69

## Supplementary materials

Further details on supplementary materials are presented online (available at https://doi.org/10.4069/kjwhn.2021.09.14).

Supplementary Table 1. Summary of literature review on studies related to breast cancer survivors
