# Supplementary material for: The development of a lifestyle modification mobile application, “Health for You” for overweight and obese breast cancer survivors in Korea
Source: Korean J Women Health Nurs. 2021 Sep 30;27(3):243–55. doi: 10.4069/kjwhn.2021.09.14 (PMC9328594; doi:10.4069/kjwhn.2021.09.14)
Supplement: Supplementary Table 1. — Summary of literature review on studies related to breast cancer survivors [file kjwhn-2021-09-14-suppl.pdf]

Supplementary Table 1. Summary of literature review on studies related to breast cancer survivors

First author (year) [reference]	Intervention content
Basen (2006) [S1]	Physical exercise for BCS
Kim (2014) [S2]	-Complex exercise program
Kim (2009) [S3]	-Cardiovascular exercise
Lyons (2016) [S4]	-Behavior program
McCarroll (2015) [S5]	-Physical activity promotion program
Park (2013) [S6]	
Park (2014) [S7]	
Quitilliani (2016) [S8]	
Unm (2017) [S9]	
Park (2009) [S10]	Psychological programs for BCS
Park (2013) [S11]	-Meditation program
Seo (2016) [S12]	-Distress, fatigue
Yoon (2014) [S13]	-Transition nursing program
	-Psychological education support program
	-Stress relief program
Fillon (2008) [S14]	Dietary interventions for BCS
Hsieh (2008) [S15]	-Importance diet management
Pierce (2007) [S16]	-Maintain normal weight
	-Weight loss diet management
DeCocker (2015) [S17]	Web and/or mobile programs for BCS
Lyons (2016) [S4]	
McCarroll (2015) [S5]	
Quintiliani (2016) [S8]	
Uhm (2017) [S9]	
Bultz (2000) [S18]	Partner, couple, marital programs for BCS
Cochrane (2011) [S19]	-Strategies to increase marital intimacy
Hoskines (2008) [S20]	
Kweon (2015) [S21]	
Lee (2011) [S22]	
Lewis (2008) [S23]	
Manne (2005) [S24]	
Naaman (2009) [S25]	
Park (2010) [S26]	
Scott (2004) [S27]	

BCS: Breast cancer survivor.
